# Defining frailty using a modified Fried’s Frailty Phenotype in a Southern African context

**DOI:** 10.1371/journal.pone.0340723

**Published:** 2026-02-04

**Authors:** E. I. Y. Madela, C. L. Gregson, F. Paruk, A. J. Burton, R. Patel, H. Wilson, F. Habana, A. M. Manyara, B. Mbanjwa, L. Gates, C. Grundy, K. A. Ward, B. Cassim

**Affiliations:** 1 Department of Geriatrics, University of KwaZulu-Natal, Durban, KwaZulu-Natal, South Africa; 2 Global Health and Ageing Research Unit, Bristol Medical School, University of Bristol, Bristol, United Kingdom; 3 Division of Internal Medicine, University of KwaZulu-Natal, Durban, KwaZulu-Natal, South Africa; 4 Musculoskeletal Research Unit, Translational Health Sciences, Bristol Medical School, University of Bristol, Bristol, United Kingdom; 5 MRC Lifecourse Epidemiology Centre, Human Development and Health, University of Southampton, Southampton, England, United Kingdom; 6 MRC International Statistics and Epidemiology Group, London School of Hygiene and Tropical Medicine, London, United Kingdom; Instituto Nacional de Geriatria, MEXICO

## Abstract

**Introduction:**

Frailty leads to disability, morbidity, and mortality in older persons. The Fried’s Frailty Phenotype (FFP), derived in the American Cardiovascular Health Study (CHS), is widely used around the world to define frailty, but lacks adaptation in African populations.

**Objective:**

To derive a modified FFP definition which best identifies frailty in a Southern African context.

**Methods:**

A population-based cross-sectional study of adults aged ≥40 years collected data from questionnaires and physical assessments. Original CHS, population-dependent, European Working Group on Sarcopenia in Older People2 (EWGSOP2) and Sarcopenia Definitions and Outcomes Consortium (SDOC) and independent thresholds were all applied to the five FFP criteria (weight loss, exhaustion, low physical activity [PA], low grip strength [GS] and slow walking speed [WS]) to assess non-differentiality, internal consistency, and plausibility.

**Results:**

The 919 participants had a median age of 59 years [IQR 50–70], 53.3% were female. Self-reported exhaustion was reported by 37.5%nd self-reported weight loss by 34.9%. Using the lowest quintile of body mass index (BMI), missed 15.2% of overweight and obese participants who reported weight loss. Using CHS thresholds, low PA was present in 36.7%. Grip strength correlated better with age (r = −0.45) than BMI (r = −0.19). Therefore, the sex-specific tenth percentile of the 40–49-years-age band of the study population was used rather than the CHS approach. The modified SDOC threshold identified slow WS in almost all (85.8%) and was therefore non-differential. The EWGSOP2 and CHS thresholds identified slow WS in 52.9% and 22.9%, respectively, compared to 34.5% using the study population’s lowest quintile.

**Conclusion:**

Culture and language sensitive questions for self-reported exhaustion and weight loss, CHS thresholds for low PA, and population dependent thresholds for GS and WS were the most suitable modifications in a Southern African setting, highlighting the need for region-specific adaptations when diagnosing frailty.

## Introduction

Life expectancy (LE) is increasing globally [1], especially in sub-Saharan Africa (SSA) where improvements in survival have added nearly 11 years to the average LE from 1990 to 2021 [1]. Reflecting this trend, in South Africa (SA) the proportion of persons aged 60 years and over increased from approximately 7.7% (3.7 million) in 2006 to 9.2% (5.6 million) in 2022 [2]. With this increase in LE a rapid rise in age-related conditions, such as frailty, is expected.

Ageing is accompanied by physiological changes across multiple systems [3], leading to a steady decline in homeostatic reserves, and increasing risk of non-communicable diseases and geriatric syndromes, including frailty. Frailty is defined clinically as an increased vulnerability towards dependency and/or death when exposed to a stressor, such as acute illness, injury, or psychological stress [4,5]. It is a major cause of disability, morbidity, and mortality in older persons [6,7] and is associated high healthcare costs [6].

The prevalence of frailty differs by geographic location, socio-economic status, and how frailty is measured [6,8–12]. Numerous clinical and research scales have been developed to assess frailty. The Fried’s Frailty Phenotype (FFP) defines frailty by its physical characteristics, or ‘phenotype’, namely the presence of three or more of: exhaustion (self-reported), shrinking (weight loss), weakness (low grip strength), slowness (slow walking speed) and low physical activity [4]. Pre-frail equates to the presence of one or two of these features. The relative simplicity of the assessment needed to define the FFP makes is feasible and practical to use in a resource-constrained setting, such as Southern Africa.

The FFP was originally derived in 2001 from two cohorts (n = 5,317) of American subjects enrolled in the Cardiovascular Health Study (CHS) [4]. It has since been successfully applied in multiple epidemiological studies globally where it is predictive of adverse clinical outcomes, including mortality [13–15]. Due to socio-cultural differences, the FFP has been modified in several countries [16–25]. An example is the use of sarcopenia thresholds from the Sarcopenia Definitions and Outcomes Consortium (SDOC) [26] and the European Working Group on Sarcopenia in Older People2 (EWGSOP2) [27] to define slow walking speed. Another example relates to mean grip strength (GS), which varies with ethnicity with lower values reported in African populations compared to European and North American populations [28], invalidating the use of CHS thresholds in a Southern African context. Hence, there have been efforts to modify the FFP for use in SSA, but none have approached this in a systematic way. Inconsistent definition modifications make prevalence comparisons difficult [16]. A recent scoping review on application of screening instruments for frailty in SSA identified 17 studies, which used seven different frailty screening methods, with only one developed and validated in SSA [29]. The brief frailty screening tool in Tanzania (BFIT) was developed in older population in a rural low-resource setting. The study population was characterized by low educational attainment, traditional lifestyles and diet, subsistence farming as the main form of employment, and limited access to healthcare services [30]. The tool has not been used in SA.

In SA, the prevalence of frailty in community-dwelling older adults ranges between 5.4% and 38% depending on frailty definitions and age range [12,17–26]. Hence, the aim of this study was to determine, from a range of different FFP modifications, the most internally consistent, differential, and plausible definition of frailty in the Southern African context.

## Materials and methods

### Study population and recruitment

This study utilised data from a population-based cross-sectional study conducted in KwaMashu, an urban area of KwaZulu-Natal, SA [31], which lies 12 kilometres north of eThekwini (Durban) and is home to 175,663 persons, 37,633 (21.4%) of whom are aged 40 years or older [2]. Recruitment began in April 2022 and ended in February 2023. Satellite images and OpenStreetMap, available through the software Aeronautical Reconnaissance Coverage Geographic Information System (ARCGIS), were used to map all of KwaMashu. Household blocks were randomly selected to be representative of the underlying population and maximise generalisability of findings. Population denominator data by sex and five-year age-bands, obtained through national census data (2011), were used to build a stratified random quota sampling frame. Participants (1 male: 1 female) were recruited by fieldworkers from households. The study aimed to recruit 168 men and 168 women in each of three age strata 40–54, 55–69 and ≥70 years, to reach a total of 504 women and 504 men. Adults were eligible if they were aged 40 or older and had lived and shared meals in the household for at least four weeks. Participants provided written or thumbprint informed consent. For those who lacked capacity to consent due to cognitive impairment, legal guardian (proxy) written/ thumbprint informed consent was sought. Pregnant women were excluded. Consenting participants were then assessed at their local clinic [31].

### Data collection and measurements

Data were collected using questionnaires (translated into isiZulu) and standardized examination forms, with data entered directly into Research Electronic Data Capture (REDCap)^®^ [32], with inbuilt data validation checks. Sociodemographic data included sex and age, verified by the date of birth on identification documents. A single question from the Shona Symptom Questionnaire (SSQ) “I felt run down” was asked to identify self-reported exhaustion [33]. A question “Have you, or those close to you, noticed that you have lost weight or become thinner in the last 12 months?” was asked to determine the presence of weight loss (because home scales are not widely available and so measured weight loss is not possible in this setting). Physical activity was assessed using the short form of the International Physical Activity Questionnaire (IPAQ) [34]. Metabolic equivalent (MET) minutes were calculated for walking, moderate intensity and vigorous intensity activities in accordance with IPAQ instructions and converted to kilocalories (Kcal) per week [35]

All measurements were performed by trained research staff, following standardised operating procedures. Grip strength (GS) was measured in kilograms (kgs) using a Jamar dynamometer (ASP Global, Atlanta Industrial Parkway, USA). Contraindications included recent (within 12 weeks) hand trauma or surgery, excessive pain, swelling or hand inflammation. Grip strength was measured three times in each hand, and the maximal GS was used. Walking speed (WS) in seconds was measured by timing participants’ walking at their usual pace over a 4-metre (13.1 feet) flat surface. Participants who used walking aids at home performed the test with their walking aids. Balance was assessed by 10 second tandem, semi-tandem and full tandem stands (not using walking aids) and chair stand time (five sit-to-stands as fast as possible) with arms folded across the chest. To derive the Short Physical Performance Battery (SPPB), WS, balance, and chair stand times were each allocated between 0 and 4 points. A score of >9–12 was regarded as normal and 0–9 as low [36]. Weight was measured in kilograms (kg) using a calibrated Seca® scale (SECA Gesellschaft mit beschränkter Haftung (GMBH) & Co., Hamburg, Germany) (participants wearing minimal clothing, shoes and socks removed, pockets emptied). Standing height was measured in millimetres (mm) using a portable Seca® stadiometer (shoes, socks, hats, head scarves removed). Mid-upper arm circumference (MUAC) was measured on the non-dominant arm using an ergonomic Seca® measuring tape; < 24 cm was considered low [37]. All measurements were repeated and the mean calculated. If there was a difference of >0.5 kgs or >5 mm between measurements, measurements were repeated until the difference was < 0.5. Body mass index (BMI) (kg/m^2^) was calculated by dividing weight (kg) by height (metres) squared. World Health Organization (WHO) categories were used to classify BMI as underweight: < 18.5 kg/m^2^, normal: 18.5–24.9 kg/m^2^, overweight: 25–29.9 kg/m^2^, obese class I: 30–34.9 kg/m^2^, obesity class II: 35–39.9 kg/m^2^ and morbidly obese (class III): ≥ 40 kg/m^2^.

### Development of diagnostic criteria for frailty

#### Original Fried frailty criteria.

**Table 1** shows the original CHS thresholds for each of the five FFP criteria. A positive response to either or both questions from the Centre for Epidemiologic Studies Depression (CES-D) Scale [38] defined exhaustion. Weight loss was defined as unintentional loss of >10 pounds in the last year, or ≥5% measured weight loss when compared to the previous year’s body weight. Physical activity was measured in kcal using the Minnesota Leisure Time Physical Activity Questionnaire (MLTPA) [39]. Low GS and slow WS were determined using CHS study-specific lowest sex-specific quintiles, stratified by BMI and height, respectively. Frailty was considered to be present if three or more criteria were met and pre-frailty if one or two criteria were met. The absence of any criteria classified the person as being robust [4]

**Table 1:** Comparison of the five FFP criteria between the original CHS and context-specific modifications in the KwaMashu study.

	Cardiovascular Health Study [CHS]: Original approach to defining FFP				KwaMashu Study: Context-specific modifications to defining FFP	Justification for context-specific modification
**Exhaustion**	Self-reported based on a positive response to either or both of the following statements from the CES–D Scale. In the last week: i) I felt that everything I did was an effort ii) I could not get going.				Positive response to a single statement from the SSQ: i) “I felt run down”	The SSQ is more culturally appropriate and a validated measure of common mental health symptoms in Southern Africa [33,40].
**Weight loss**	Self-reported loss of >10 pounds unintentionally in the last year, or ≥5% measured weight loss when compared to previous year’s body weight				i) Have you, or those close to you, noticed that you have lost weight or become thinner in the last 12 months? ii) Lowest sex-specific quintile of BMI	Measuring weight is not common in this population. Access to scales is limited and routinely collected baseline weight was not available. Hence, we took a pragmatic approach of ‘noticeable weight loss’, by the participant or someone close to them. Lowest sex-specific quintile of BMI as used in previous studies [16–18,22,25]
**Low physical activity**	Self-reported: MLTPA. Kilocalories (Kcal) of activity per week were estimated. i) Lowest 20% identified as meeting the frailty criteria. Men: <383 Kcals/week Women: <270 Kcals/week				Self-reported: IPAQ Short Form. Metabolic equivalent (METS) minutes/week calculated and converted to Kcal/per week. i) Same CHS thresholds ii) Lowest 20% of the KwaMashu study stratified by sex	The IPAQ Short Form has been validated in SA [41], the MLTPA was not contextually appropriate.
**Low grip strength**	Maximal grip strength of three measures stratified by sex and BMI. i) Lowest 20% identified as meeting the frailty criteria.				Maximal grip strength of six measures. i) CHS thresholds ii) Lowest 20% of the KwaMashu study 40-49-year age band stratified by sex iii) Lowest 10% of the KwaMashu study 40-49-year age band stratified by sex.	Normative data or appropriate reference range were not available for Southern Africa.
	Men		Women			
	BMI (kg/m^2^)	GS (kg)	BMI (kg/m^2^)	GS kg		
	≤24	≤29	≤23	≤17		
	24.1–26	≤30	23.1–26	≤17.3		
	26.1–28	≤30	26.1–29	≤18		
	>28	≤32	>29	≤21		
**Slow walking speed**	Measured time to walk 15 feet. i) Lowest 20% of CHS stratified by sex and median height, identified as meeting the frailty criteria.				Mean measured time of two 4-metre (13.1 feet) walks. i) CHS thresholds ii) Lowest 20% of the KwaMashu study stratified by sex and median standing height. iii) Modified SDOC (<1m/s) iv) EWGSOP2 (<0.8 m/s)	Normative data or appropriate reference range were not available for Southern Africa. The EWGSOP2 thresholds have been used previously in SA [42,43].
	Men: ≤173cm: ≥7 s (0.7 m/s)					
	>173cm: ≥6 s (0.8 m/s)					
	Women: ≤159cm: ≥7 s (0.7 m/s)					
	>159cm: ≥6 s (0.76 m/s)					

Abbreviations: BMI: body mass index, CES–D: Centre for Epidemiologic Studies Depression Scale, cm: centimetre, EWGSOP2: European Working Group on Sarcopaenia2, IPAQ: International Physical Activity Questionnaire, Kcals/week: Kilocalories per week, kg: kilogram, kg/m^2^: kilograms per metre squared, MET-min: Metabolic equivalent minutes, m/s: metres per second, MLTPA: Minnesota Leisure Time Physical Activity Questionnaire, s: second; SDOC: Sarcopaenia Definition and Outcomes Committee

#### Assessment of frailty and context-specific modifications to the FFP CHS criteria.

In the current study (KwaMashu study) the following modifications were applied to the original FFP criteria (CHS study).

1.
**Exhaustion**

A positive response to a single statement from the SSQ “I felt run down” was used to identify exhaustion.

2.
**Weight loss**

To quantify unintentional weight loss participants were asked: “Have you, or those close to you, noticed that you have lost weight or become thinner in the last 12 months?”. Due to concerns over recall bias, we further considered defining weight loss based on the lowest sex-specific quintile of BMI.

3.
**Physical activity**

The IPAQ Short Form [34], calculated MET minutes/week, which were converted to Kcal per week. The threshold for defining low PA used in the CHS (the sex-specific lowest quintile) was compared to the sex-specific lowest quintile in the KwaMashu population.

4.
**Grip strength**

For GS, the following were used as potential thresholds: (i) the lowest sex-specific quintile of CHS GS stratified by BMI, (ii) the lowest sex-specific quintile of GS in the KwaMashu study’s youngest age band (40–49 years) and (iii) the lowest sex-specific 10^th^ percentile of GS in the KwaMashu study’s youngest age band (40–49 years).

5.
**Walking speed**

Four different thresholds were applied to potentially identify slow WS (Table 1), based on the existing study population and international guidelines developed for the diagnosis of sarcopaenia, the: (i) original FFP CHS threshold, ii) lowest sex-specific KwaMashu study quintile stratified by median standing height, (iii) 2023 modified SDOC threshold of <1 m/s [26], and (iv) <0.8 m/s as recommended by the EWGSOP2 [27].

As per the original FFP, the presence of three or more criteria classified the participants as frail, one or two as pre-frailty and the absence of all criteria indicated someone was robust.

### Ethical and governance approvals

Ethical approval was obtained from the University of KwaZulu-Natal’s Biomedical Research Ethics Committee; reference numbers BREC/00002513/2021and BREC/00005591/2023. Permission to conduct the study was granted by the Health Committee of the local area council and the District and Provincial Departments of Health (NHRD Ref: KZ_202106_024).

### Statistical analysis

Statistical analysis was performed with Stata® SE version 17. Data were assessed for normality using histograms. Participant characteristics were summarised using descriptive statistics; continuous data were summarised using means with standard deviation (SD) if normally distributed, or medians with interquartile ranges (IQR) if not normally distributed. Categorical values were summarised using frequency counts and percentages. Participants with missing data required to calculate FFP criteria were excluded from analyses. The percentage of participants meeting each criterion, using the different thresholds, was reported.

The appropriateness of the different approaches at defining the FFP criteria were sequentially assessed as follows; a threshold needed to (i) be differential (i.e., not be ubiquitous and not rare, and instead be able to identify a proportion of the study population >5% or < 80%), (ii) demonstrate internal consistency by correctly identify individuals, in whom other characteristics, known to be associated with frailty, are associated, and (iii) be clinically plausible. For example, to assess internal consistency, self-reported weight loss and BMI were cross tabulated and the relationships between GS, age and BMI were assessed by linear regression. Frail participants were expected to be older, with lower PA, GS, WS, BMI, MUAC, and SPPB scores compared to robust participants [4,44,45], thus these associations were investigated.

## Results

### Characteristics of the study population

In total, 5,136 households were approached; 1,440 people aged ≥40 years were identified of whom 1,035 were invited and accepted to participate, and 968 then consented and were recruited into the study. Of these 919 (94.9%) had complete data to permit analyses (S1 Fig). Their median [IQR] age was 59 [50–70] years, and 52.8% were female (Table 2). Females had a higher median BMI and MUAC than males 34.1 kg/m^2^ [28.5–39.9] vs. 24.3 kg/m^2^ [21.0–28.8], and 28.0 cm [24.4–33.4] vs. 24.6 cm [21.0–28.3] respectively, with a lower median SPPB score 9.5 [7–11] vs. 11 [8–12].

**Table 2:** Characteristics of the study population in KwaMashu, KwaZulu-Natal, South Africa.

Characteristic	Total n = 919	Men n = 434 (47.2%)	Women n = 485 (52.8%)
Age (years) (median [IQR])	59 [50-70]	59 [50-68]	60 [51-70]
Age groups, n (%)			
40-49 years	208 (22.6)	104 (24.0)	104 (21.4)
60−59 years	259 (28.2)	128 (29.5)	131 (27.0)
60-69 year ≥ 70 years	200 (21.8) 252 (27.4)	100 (23.0) 102 (23.5)	100 (20.6) 150 (30.9)
Self-reported falls, n (%)	145 (15.8)	59 (13.6)	86 (17.7)
Self-reported exhaustion, n (%)	345 (37.5)	149 (34.3)	196 (40.4)
Self-reported weight loss, n (%)	321 (34.9)	144 (33.2)	177 (36.5)
Weight (kg) (median [IQR])	76.3 [63.4-89.7]	68.7 [60.2-81.3]	82.8 [69.9-96.9]
Height (cm) (median [IQR])	161.6 [155.7-168.6]	168.6 [163.7-172.4]	156.2 [152.4-160.0]
MUAC, n (%)			
Normal (≥ 24 cm)	617 (67.1)	241 (55.5)	376 (77.5)
Malnutrition (<24 cm)	300 (32.6)	191 (44.0)	109 (22.5)
Missing	2 (0.2)	2 (0.5)	
MUAC (cm) (median [IQR])	26.4 [22.0-31.0]	24.6 [21.0-28.3]	28.0 [24.0-33.4]
BMI (kg/m^2^)(median [IQR])	28.9 [23.4-35.4]	24.3 [21.0-28.8]	34.1 [28.5-39.9]
BMI categories (kg/m^2^), n (%)			
Underweight: < 18.5	44 (4.7)	37 (8.5)	7 (1.4)
Normal: 18.5–24.9	262 (28.5)	207 (47.7)	55 (11.3)
Overweight: 25–29.9	185 (20.1)	100 (23.0)	85 (17.5)
Obese class I: 30–34.9	183 (19.9)	67 (15.4)	116 (23.9)
Obesity class II: 35–39.9	121 (13.2)	19 (4.4)	102 (21.0)
Morbidly obese (class III): ≥ 40	124 (13.5)	4 (0.9)	120 (24.7)
Grip strength (kg) (median [IQR])	32 [26-38]	38 [32-44]	28 [22-33]
Walking speed in m/s (median [IQR])	0.8 [0.6-0.9]	0.8 [0.7-1.0]	0.7 [0.6-0.9]
Physical activity (Kcal/ week) (median [IQR])	399.8 [0-2273.1]	726 [0-2556.1]	197.6 [0-1654.8]
SPPB score (median [IQR])	10 [8-11]	11 [8-12]	9.5 [7-11]
SPPB			
Normal (score >9–12) n (%)	497 (54.1)	267 (61.5)	230 (47.4)
Low (score 0–9) n (%)	390 (42.4)	160 (36.9)	230 (47.4)
Missing	32 (3.5)	7 (1.6)	25 (5.2)

Abbreviations: BMI: body mass index, cm: centimetre, IQR: Interquartile range, Kcal/week: Kilocalories per week, kg: kilogram, kg/m^2^: kilograms per metre squared, MUAC: Mid-upper arm circumference, SPPB: Short Physical Performance Battery

### Criteria for frailty: proportions using different thresholds

1.
**Exhaustion**

The prevalence of self-reported exhaustion was 37.5% (n = 345); with 34.3% (n = 149) males and 40.4% (n = 196) females reporting feeling run down (Table 2).

2.
**Weight loss**

Overall 34.9% (n = 321) self-reported weight loss, whilst 19.7% (n = 181) fell within the lowest sex-specific quintile of BMI (<20.4 kg/m^2^ in men and <27.4 kg/m^2^ in women) and 15.2% were either overweight or obese (Table 3). Only 7.5% (n = 24) participants self-reporting weight loss were underweight, with the majority being either overweight (21.1%; n = 71) or obese (38.9%, n = 125), suggesting that using BMI alone missed weight loss in this population, thus self-reported weight loss was used (S1 Table).

**Table 3:** The proportion of the study population with each of the five components of frailty, according to the different thresholds applied.

Frailty criteria	Components				n (%)
**Exhaustion**	Self-reported: Positive response to a single statement from The Shona Symptom Questionnaire “I felt run down”				345 (37.5)
**Weight loss**	i. Have you, or those close to you, noticed that you have lost weight or become thinner in the last 12 month				321(34.9)
	ii. Lowest sex-specific quintile of BMI (men: < 20.4 kg/m^2^, women <27.4 kg/m^2^)				181 (19.7)
**Low Physical Activity**	i. Lowest sex-specific quintile of the CHS population (men: < 383 Kcals/week, women: < 270 Kcals/week)				337(36.7)
	ii. Lowest sex-specific quintile of the study cohort				Not quantifiable
**Low Grip Strength**	i. Lowest sex-specific quintile CHS study stratified by BMI.				188 (20.5)
	Men		Women		
	BMI (kg/m^2^)	GS (kg)	BMI (kg/m^2^)	GS (kg)	
	≤ 24	≤ 29	≤ 23	≤ 17	
	24.1–26	≤ 30	23.1–26	≤ 17.3	
	26.1–28	≤ 30	26.1–29	≤ 18	
	> 28	≤ 32	> 29	≤ 21	
	ii. Lowest sex-specific quintile of the 40–49 years age band of the KwaMashu study (Men: < 36 kg Women: < 28 kg)				484(52.7)
	iii. Lowest sex-specific 10^th^ percentile of the 40–49 years age band of the KwaMashu study (Men: < 32 kg Women: < 22 kg)				280 (30.5)
**Slow Walking Speed**	i. Slowest sex-specific quintile of the CHS population stratified by median standing height				210(22.9)
	Men		Women		
	≤ 173 cm: ≥ 7 s (0.7 m/s)		≤ 159 cm: ≥ 7 s (0.7 m/s)		
	> 173 cm: ≥ 6 s (0.8 m/s)		> 159 cm: ≥ 6 s (0.8 m/s)		
	ii. Slowest sex-specific quintile of the KwaMashu study stratified by median standing height.				317(34.5)
	Men		Women		
	≤ 168.2 cm: ≥ 5.3s (0.8 m/s)		≤ 156.5 cm: ≥ 6.6s (0.6 m/s)		
	> 168.2 cm: ≥ 5.8s (0.7 m/s)		> 156.5 cm: ≥ 5.4s (0.7 m/s)		
	iii. < 1 m/s (modified SDOC)				788 (85.8)
	iv. < 0.8 m/s (EWGSOP)				486 (52.9)

Abbreviations: BMI: body mass index, CHS: Cardiovascular Health Study, cm: centimetre, EWGSOP: European Working Group on Sarcopaenia, FFP: Fried Frailty Phenotype, GS: Grip strength, Kcals/week: Kilocalories per week. kg: kilogram, kg/m^2^: kilograms per metre squared, m/s: metres per second, PA: Physical activity, s: second, SDOC: Sarcopaenia Definition and Outcomes Committee, WS: Walking speed.

3.
**Low physical activity**

Physical activity consumed and estimated median [IQR] of 726.2 [0–2556.1] and 197.6 [0–1654.8] Kcal per week in men and women, respectively. Almost half (49%) of study participants reported not walking for more than 10 minutes a day in the last week, thus both the lowest and second lowest quintiles were characterized by (almost) zero minutes of PA in a week, invalidating an approach based on the lowest quintile. Therefore, the CHS thresholds (lowest of sex-specific quintiles) were applied, which classified 36.7% of participants as having low PA (Table 3).

4.
**Low grip strength**

The median [IQR] grip strength was 38 [32−44] kg for males and 28 [22−33] kg for females. Using the sex-specific CHS thresholds, stratified by BMI, low GS was prevalent in 20.5%. Given the high prevalence of obesity, the relationship between GS and BMI was investigated to internally validate the CHS approach; however, we found a poor correlation (overall r = −0.19, r = 0.14 in males and r = 0.09 in females), invalidating the CHS sex-specific, BMI-stratified approach. Instead, GS was more closely correlated with age (overall r = −0.45, r = −0.42 in males and r = −0.56 in females) (S2 Fig), supporting the use of sex-specific age-stratified thresholds. The lowest sex-specific quintile (20%) of GS in the KwaMashu study’s 40–49-year age group categorized 52.7% as having low GS, while 30.5% met the criterion when a 10^th^ percentile threshold was used (Table 3). The 10^th^ percentile (which was also equivalent to ≤2 SDs below the mean) was favoured over the lowest quintile approach as it is more clinically plausible for the younger age group to have good GS, with only 10% at most having low GS.

5.
**Slow walking speed**

The median [IQR] WS was 0.8m/s [0.7–1.0] and 0.7m/s [0.6–0.9] m/s for men and women respectively. Almost all of the study population met the criterion for slow WS (85.8%) when the modified SDOC threshold of <1.0 m/s was used; suggesting this was a non-differential approach. Using the CHS WS thresholds, a lower than seemed clinically plausible prevalence of slow WS (22.9%) was identified. The lack of applicability of the CHS approach may be explained by the shorter height of the KwaMashu study population compared to the CHS population (168.2 cm vs 173 cm for males and 156.5 cm vs 159 cm for females respectively).

Using the KwaMashu study population’s sex-specific lowest quintile, stratified by median height, 34.5% had a slow WS, whilst 52.9% did when using the EWGSOP2 thresholds. As both were clinically plausible, both these thresholds were then used in two frailty modifications for further comparison.

### Frailty modifications

The prevalence of individual frailty criteria within the two remaining potential FFP modifications, differing only by the WS threshold used, are shown in Supplementary Table 2, with mean age, anthropometric characteristics, and SPPB scores summarised in (Table 4). In both modifications, exhaustion and weight loss were self-reported, CHS thresholds defined low PA, and the sex-specific 10^th^ percentile of the 40–49 years age group of the KwaMashu study defined low GS. In the first modification the EWGSOP2 threshold was applied to WS, generating prevalence of pre-frailty and frailty of 57.7% and 29.7% respectively. In the second modification low WS speed was defined as the KwaMashu population’s sex-specific lowest quintile stratified by median height; this generated prevalence of prefrailty and frailty of 58.2% and 25.5%, respectively, so the modifications were similar in this regard (S2 Table).

**Table 4:** Distribution of mean age, anthropometric measures, SPPB scores and frequency of frailty criteria according to the two potential FFP modifications.

			Objective measures						Self-reported measures		
	Number of frailty criteria	% distribution	Mean Age (years)	Mean BMI (Kg/m^2^)	Mean MUAC (cm)	Mean SPPB score	Mean Walking speed (s)	Mean Grip strength (kg)	Weight loss n %	Exhaustion n %	Mean Kilocalories (Kcals/week)
**Modification 1**	0	116 (12.6)	54.1	28.7	27.4	10.9	4.2	40.0	0	0	3931.0
	1	261 (28.4)	56.4	29.7	27.3	10.1	4.9	36.4	43 (13.4)	58 (16.8)	5413.1
	2	269 (29.3)	59.2	30.9	27.0	9.1	5.8	31.8	97 (30.2)	102 (29.6)	3633.8
	**3**	**156 (17.0)**	**64.2**	**30.8**	**26.6**	**7.9**	**6.9**	**28.0**	**85 (26.5)**	**91 (26.4)**	**1875.1**
	**4**	**83 (9.0)**	**68.6**	**30.4**	**24.9**	**6.3**	**7.3**	**24.4**	**62 (19.3)**	**60 (17.4)**	**795.1**
	**5**	**34 (3.7)**	**72.6**	**29.5**	**23.8**	**6.0**	**7.4**	**20.9**	**34 (10.6)**	**34 (9.9)**	**9.3**
**Modification 2**	0	150 (16.3)	55.5	29.5	27.8	10.6	4.4	39.3	0	0	3781.3
	1	288 (31.3)	57.1	29.9	27.1	10.1	4.9	35.1	59 (18.4)	70 (20.3)	5360.4
	2	247 (26.9)	59.4	30.6	27.0	9.0	5.8	31.1	100 (31.2)	112 (32.5)	3092.0
	**3**	**144 (15.7)**	**65.0**	**30.6**	**26.0**	**7.7**	**7.0**	**28.0**	**88 (27.4)**	**91 (26.4)**	**1829.0**
	**4**	**64 (7.0)**	**68.3**	**30.4**	**24.7**	**6.0**	**7.7**	**23.2**	**48 (15.0)**	**46 (13.3)**	**877.1**
	**5**	**26 (2.8)**	**74.0**	**30.2**	**24.0**	**5.3**	**7.8**	**21.8**	**26 (8.1)**	**26 (7.5)**	**12.2**

**Abbreviations:** BMI: body mass index, cm: centimetres, kg: kilograms, Kg/m^2^: kilograms per metre squared, Kcals/week: Kilocalories per week, MUAC: Mid-upper arm circumference, SPPB: Short Physical Performance Battery, s: seconds.

Modification 1: Self-reported exhaustion and weight loss, CHS thresholds for low PA, sex-specific 10th percentile of the 40–49 years age group of the KwaMashu study for low GS and EWGSOP2 (0.8 m/s) threshold for slow WS.

Modification 2: Self-reported exhaustion and weight loss, CHS thresholds for low PA, sex-specific 10^th^ percentile of the 40–49 years age group of the KwaMashu study for low GS and KwaMashu population’s sex-specific lowest quintile stratified by median height threshold for slow WS.

**Fig 1:**
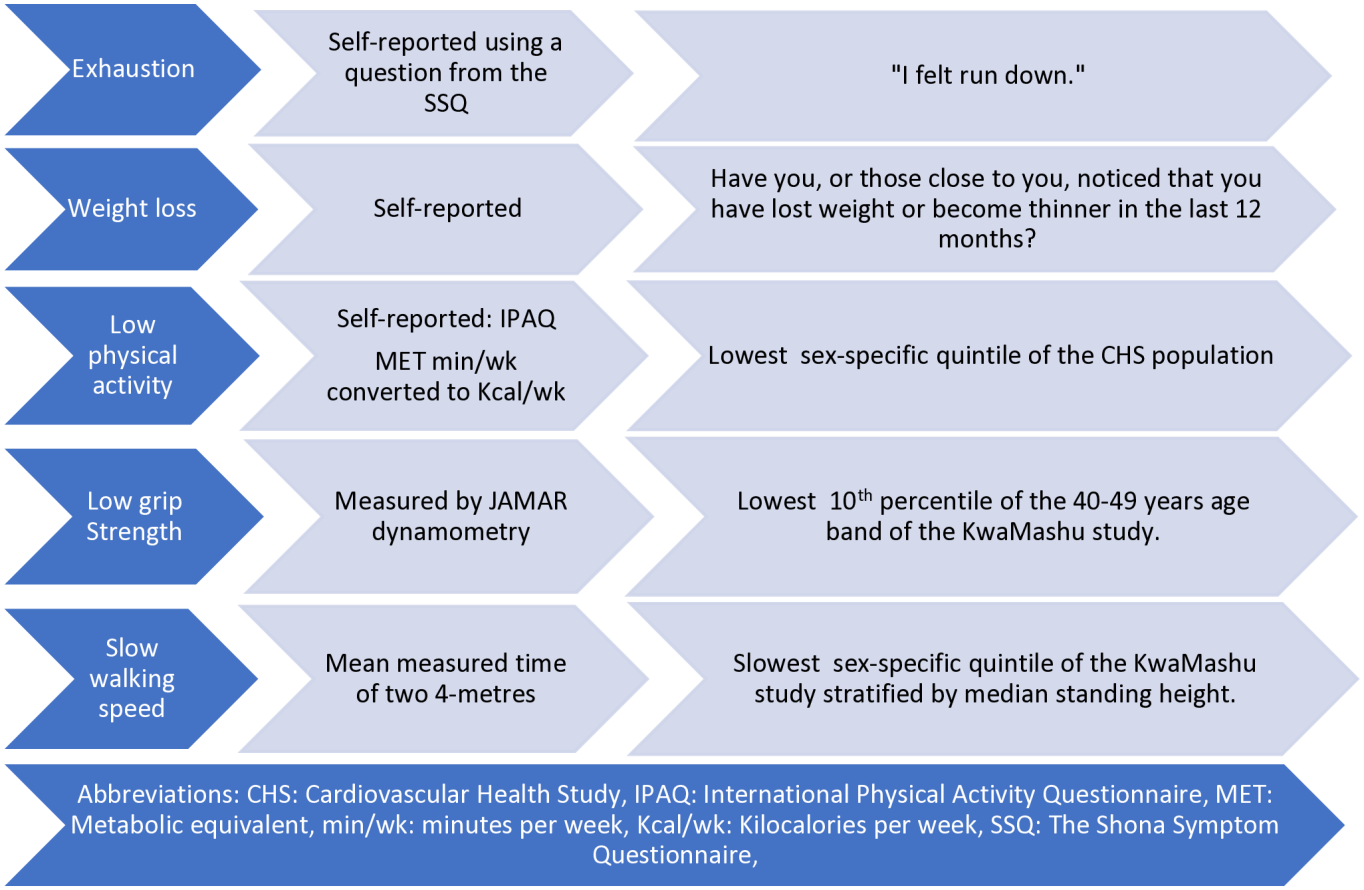
Summary of the most suitable modifications to the five FFP criteria in urban South Africa.

When comparing the two modifications, frail participants in modification two were older, with lower SPPB score, lower MUAC and took longer to walk over the 4-meters as compared to modification one. Interestingly in both modifications, there was no consistency between the number of frailty criteria and BMI. For consistency we opted to use the study specific threshold for WS in the final model, namely modification 2, as summarized in Fig 1.

## Discussion

The purpose of this study in KwaMashu, SA, was to determine the most suitable modifications and thresholds for FFP criteria in the local population. We modified the FFP criteria for frailty by using culturally and linguistically appropriate questions for exhaustion and weight loss. For PA, although we used the IPAQ rather than the MLTPA questionnaire to derive kcal/week, we used the CHS threshold, whereas for GS and WS population-dependent thresholds were applied. Thus, the modified FFP, mostly using population specific thresholds, was identified as the most suitable, internal consistent modification in this setting.

The original FFP criteria and thresholds, derived in the CHS study using their population specific thresholds, have been validated in high income countries. However, these criteria and thresholds are influenced by ethnic variations, socio-cultural differences, levels of health awareness and poverty, triggering the need for modifications in SSA [8,43,46–49]. A prerequisite of the translation of questionnaires developed in English into local languages (in this instance IsiZulu) is that the original meaning and intent of questions is maintained. However, a previous study in SA found that translating one of the questions from the CES-D used to denote exhaustion in the original FFP, (“everything you did was an effort”) was found to be ambiguous [43]. We therefore opted to use a more culturally and linguistically appropriate question from the SSQ which has been validated in this setting to detect common mental health symptoms, including exhaustion [40,50].

Quantifying weight loss is uncommon in our population, due to a lack of health awareness, non-availability of weighing-scales, as well as differences in appreciation of body image [51]. While BMI was explored as a potential as a marker of weight loss, weight loss was masked by the high prevalence of obesity [52,53]. A more suitable alternative in this context was self-reported weight loss significant enough to be noticed by those closest to the participant. Although intentional weight loss is uncommon currently, this may change in the future in response to public health interventions. In which case, the question will need to be revised to capture unintentional weight loss specifically.

Quantifying PA in the KwaMashu population was challenging even when substituting the MLTPA questionnaire (used in the CHS) with the IPAQ – Short Form which has been validated in SA [41]. Despite this contextual validation, vigorous and moderate activities like heavy lifting, digging, fast bicycling, are activities that may require access to equipment or a gym subscription, which are uncommon practices for older persons in this resource constrained setting. Surprisingly, 49% of the study population reported not walking for more than 10 minutes in the past week, scoring 0 Kcals/week, which suggests that the IPAQ may not be sensitive enough to capture minimal to mild activity, as most participants reported no disability or walking difficulties. We were therefore unable to use the lowest quintile of the KwaMashu study population as a threshold for low PA and defaulted to use the CHS thresholds instead.

Grip strength, as a measure of muscle strength, decreases with age and is associated with disability and increased mortality [54,55]. Ethnic variations in GS invalidate the use of CHS thresholds [28]. Furthermore, the normative data derived for African populations in the Prospective Urban Rural Epidemiology (PURE) study have an upper age limit of 70 years and were therefore not appropriate for this study [28]. The CHS thresholds for low GS were stratified by BMI. There is conflicting evidence of the relationship between GS and BMI, potentially as BMI is unable to distinguish between muscle and fat components of body composition. In an Australian study greater GS was weakly related to higher BMI in adults under the age of 30 years and those over the age of 70 years, but inversely related to higher BMI between these ages [56]. In India, Dhananjaya et al noted that BMI and GS were negatively correlated in male participants with normal BMI and weakly negatively correlated in obese males. In overweight females, GS and BMI were weakly negatively correlated, but not in those with normal BMI [57]. Thus BMI might influence the interpretation of low GS in countries with high levels of obesity such as SA. We found that in our population where the prevalence of obesity is high, age correlated better with GS than did BMI, justifying using the lowest sex-specific 10^th^ percentile of the 40–49 years age group as the threshold rather than BMI based approach.

Walking speed, while easy to measure and an important indicator of poor health outcomes in older persons, is affected by several factors including age, lower extremity muscle power and leg-length [58]. Thus, WS thresholds were stratified by sex and median standing height in the CHS. Compared to the CHS, the SA participants were shorter (173 cm vs 168.2 cm and 159 cm vs 156.5 cm, for males and females, respectively), invalidating the use of the CHS threshold in this setting. Using WS thresholds recommended by SDOC and EWGSOP (mostly derived from high-income populations) of <1m/s and <0.8m/s, respectively [26,27], overestimated the prevalence of slow WS in this setting, highlighting the need for population-specific thresholds. We therefore adopted the slowest sex-specific quintile of the KwaMashu study population stratified by median standing height.

In a systematic review of 262 studies investigating the impact of modifications to the FFP on frailty classification, prevalence, and predictive ability, modifications were commonly made to weight loss and low PA definitions [16]. Experiencing similar limitations when quantifying weight loss, studies have used either the lowest quintile of BMI for their population, or a BMI < 18.5 kg/m^2^ to define weight loss [16–18,22,25]. This approach was not effective in our setting where SA is within the top 10 countries with the highest levels of obesity [53]. The prevalence of obesity in SA was reported to be 32.1%, 15.3% in males and 41.3% in females in 2023 with KZN having the highest obesity prevalence compared to other provinces [59]. Obesity is common in other SSA countries such as Gabon, Botswana, Lesotho and Swaziland [53]. Using low BMI as a marker of weight loss underreported prefrailty and frailty in SA. This was supported by the lack of a relationship between self-reported weight loss and BMI and the lack of an inverse relationship between BMI and the number of frailty criteria in our study. More participants reported weight loss, than had low BMI, supporting our rationale that weight loss observed by the participant or someone close to them was differential in identifying frailty. Findings were similar for MUAC, where more participants reported weight loss than had low MUAC. Hence, in the absence of an objective measurement of weight-change, we recommend the use of self-reported weight loss rather than BMI in this setting.

The prevalence of frailty differed in our study population depending upon the modifications used to define the five criteria of FFP, as has been reported previously [16]. The prevalence of frailty was 25.5% using the final modification. This prevalence was significantly higher than in the previous studies conducted in SA, using the FFP [8,43]. The Study on global AGEing and adult health (SAGE) reported a prevalence of 14.5% in persons aged 50 years and older [8] the Health and Aging in Africa: Longitudinal Studies of an INDEPTH Community study (HAALSI) conducted in a rural population aged 40 years and above reported a prevalence between 5.4% and 13.2% [43]. The higher prevalence in our study, may reflect a higher proportion of older people, as well as the definitions of frailty.

To our knowledge, this is first study to explore the challenges and necessary modifications to the FFP definition of frailty in a Southern African context. Recruitment was designed to be able to study a representative sample of the general population. Our study had several limitations. The insensitivity of the IPAQ to capture mild PA limited application of different thresholds to identify those with low PA. The median age of our study population was younger than the original FFP validation study. This reflects the lower average life expectancy of SA at 65 years, compared to 77 years in the United States in 2001. Even though the African population is relatively younger (chronological age), African populations tend to age faster biologically than in European populations [60]. Hence, the SAGE and HAALSI studies on frailty conducted in SA were conducted in populations aged 50 years and over and aged 40 years and over, respectively [8,43]. Our approach gave us the opportunity to compare our findings with these previous studies. Self-reported weight loss was most suitable in this setting as low BMI and/ or MUAC were not sensitive markers of weight loss due to high obesity prevalence. The intention to lose weight was not explored in this study, therefore, self-reported weight loss may have included those with healthy weight loss and overestimated the frailty prevalence. Future studies using self-reported weight loss should consider adding a question on intent. Data were all cross-sectional, so we were unable to validate the FFP definition against longitudinal outcomes such as hospital admission and mortality. Furthermore, we lacked access to electronic health records, that might have enabled computation of an electronic frailty index, against which could have validated our approach. The FFP thresholds proposed remain to be validated and in other provinces and ethnic groups in Southern Africa.

## Conclusion

In conclusion, with an increasing longevity in SA, the prevalence of frailty is likely to increase and contribute to disability, morbidity, and mortality in older persons with associated high healthcare costs. Frailty can be assessed in a number of ways and the FFP is one of the most widely used phenotypic approaches; however, contextual application is needed. We have identified thresholds for each of the five FFP criterion that are suitable for the Southern African context. These thresholds show that one in four older, urban-dwelling South African men and women are frail.

## Supporting information

S1 Fig Participant flow chart describing household-based identification and recruitment of study participants. (TIF)

S2 Fig Relationship between body mass index and grip strength and age and grip strength in the total population and in men and women. Relationship between body mass index and grip strength in the total cohort (A), in men (C) and women (D) and age and grip strength in the total population (B) and in men (E) and women (F).
(TIF)

S1 Table Body Mass Index in the participants who self-reported weight loss. (DOCX)

S2 Table The prevalence of frailty according to the three modifications of the FFP. (DOCX)

S3 Table Modified scale questionnaire. (DOCX)

S4 Table Study questionnaire. (DOCX)

S5 Table Study clinical examination. (DOCX)
