# Supplementary figures and images for: Defining frailty using a modified Fried’s Frailty Phenotype in a Southern African context
Source: PLoS One. 2026 Feb 4;21(2):e0340723. doi: 10.1371/journal.pone.0340723 (PMC12872031; doi:10.1371/journal.pone.0340723)

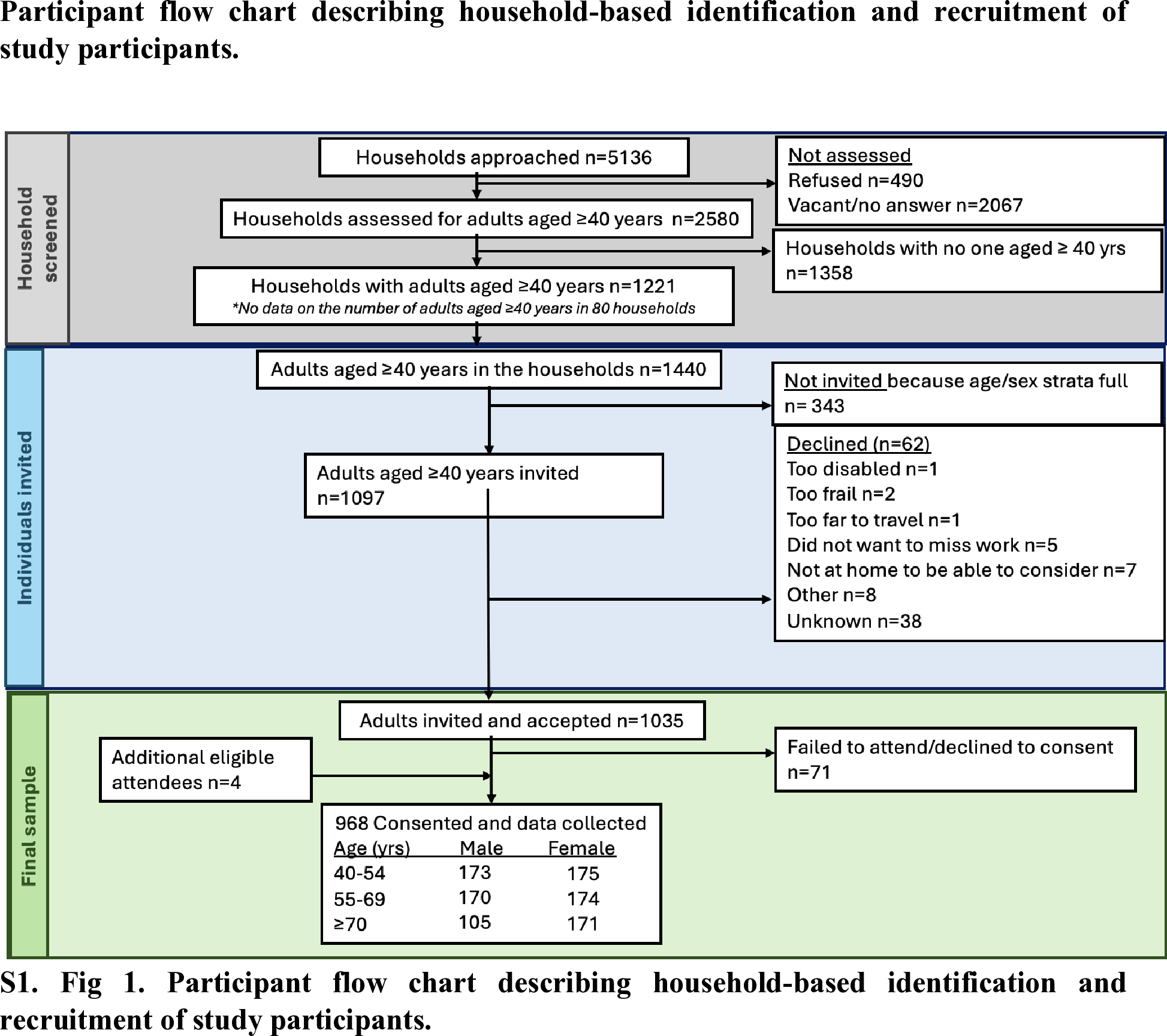

Supplement: S1 Fig — (TIF) [file pone.0340723.s001.tif]

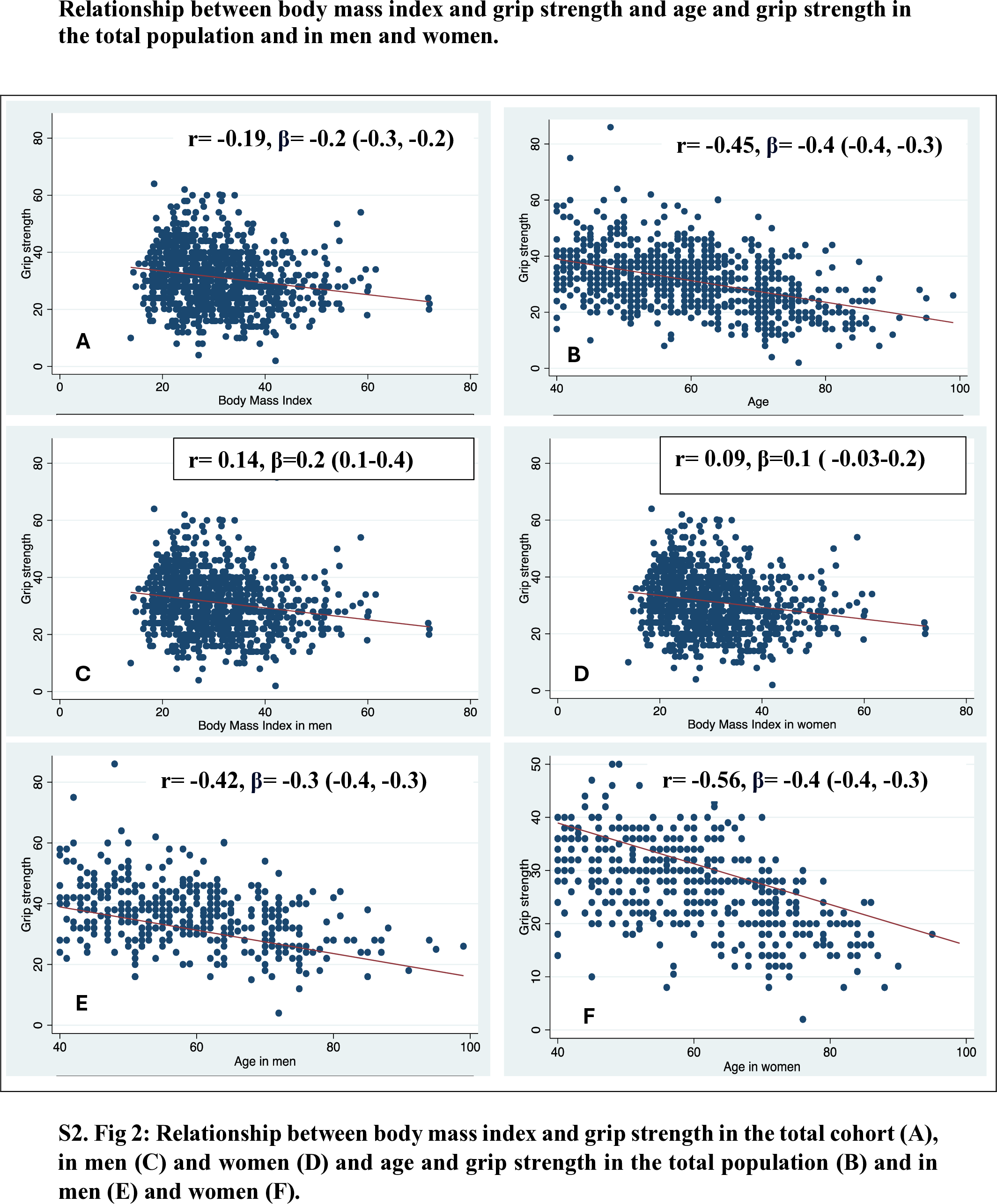

Supplement: S2 Fig — Relationship between body mass index and grip strength in the total cohort (A), in men (C) and women (D) and age and grip strength in the total population (B) and in men (E) and women (F). (TIF) [file pone.0340723.s002.tif]
