# Supplementary material for: Defining frailty using a modified Fried’s Frailty Phenotype in a Southern African context
Source: PLoS One. 2026 Feb 4;21(2):e0340723. doi: 10.1371/journal.pone.0340723 (PMC12872031; doi:10.1371/journal.pone.0340723)
Supplement: S1 Table — (DOCX) [file pone.0340723.s003.docx]

| **S1 Table : Body Mass Index in the participants who self-reported weight loss** | |
| --- | --- |
| **BMI category (kg/m^2^)** | **n (%)** |
| Underweight <18.5 | 24 (7.5) |
| Normal 18.5- <25.0 | 101 (31.5) |
| Overweight 25- <30 | 71 (22.1) |
| Obese class 1 30- <35 | 64 (19.9) |
| Obese class 2 35- <40 | 28 (8.7) |
| Obese class 3 ≥40 | 33 (10.3) |
| **Total** | **321 (100)** |

**Abbreviations:** BMI: body mass index, Kg/m^2^: kilograms per metre squared.
