# Supplementary material for: Defining frailty using a modified Fried’s Frailty Phenotype in a Southern African context
Source: PLoS One. 2026 Feb 4;21(2):e0340723. doi: 10.1371/journal.pone.0340723 (PMC12872031; doi:10.1371/journal.pone.0340723)
Supplement: S2 Table — (DOCX) [file pone.0340723.s004.docx]

| **S2 Table: The prevalence of frailty according to the three modifications of the FFP** | | |
| --- | --- | --- |
|  | **Modification 1** | **Modification 2** |
| **Self-reported exhaustion**  “I felt run down.” | 345 (37.5) | 345 (37.5) |
| **Weight loss**  “Have you, or those close to you, noticed that you have lost weight or become thinner in the last 12 months?” | 321 (34.9) | 321 (34.9) |
| **Low physical activity**  Lowest quintile of the CHS study stratified by sex | 337 (36.7) | 337 (36.7) |
| **Low grip strength**  Lowest 10^th^ percentile of the 40-49 year age group of the KwaMashu study stratified by sex | 280 (30.5) | 280 (30.5) |
| **Slow walking speed** | 486 (52.9) | 317 (34.5) |
| **Threshold for slow WS** | Slow walking speed: <0.8 m/s (EWGSOP2) | Slowest quintile of the KwaMashu study stratified by sex and median standing height |
| **Prevalence of frailty** | | |
| **Robust** | 116 (12.6) | 150 (16.3) |
| **Pre-frail** | 530 (57.7) | 535 (58.2) |
| **Frail** | 273 (29.7) | 234 (25.5) |

**Abbreviations:** CHS: Cardiovascular Health Study, EWGSOP2: European Working Group on Sarcopaenia2, FFP: Fried Frailty Phenotype.
