# Supplementary material for: Defining frailty using a modified Fried’s Frailty Phenotype in a Southern African context
Source: PLoS One. 2026 Feb 4;21(2):e0340723. doi: 10.1371/journal.pone.0340723 (PMC12872031; doi:10.1371/journal.pone.0340723)
Supplement: S3 Table — (DOCX) [file pone.0340723.s005.docx]

| **S3 Table: Modified Fried Frailty Phenotype Questionnaire** | |
| --- | --- |
| **Exhaustion** | Positive response to a single statement from the Shona Symptom Questionnaire:  “I felt run down” |
| **Weight loss** | Have you, or those close to you, noticed that you have lost weight or become thinner in the last 12 months? |
| **Low physical activity** | Self-reported: The International Physical Activity Questionnaire (IPAQ) Short Form. Metabolic equivalent (METS) minutes/week calculated and converted to Kcal/per week.  Men: <383 Kcals/week  Women: <270 Kcals/week |
| **Low grip strength** | Maximal grip strength of six measures.  Lowest 10% of the study population’s 40-49-year age band stratified by sex.  Men:<32 kg  Women: <22 kg |
| **Slow walking speed** | Mean measured time of two 4-metre (13.1 feet) walks.  Lowest 20% of the KwaMashu study stratified by sex and median standing height  Men: ≤ 168.2 cm: ≥5.3s (0.8 m/s)  > 168.2 cm: ≥5.8s (0.7 m/s)  Women: ≤ 156.5 cm: ≥6.6s (0.6 m/s)  > 156.5 cm: ≥5.4s (0.7 m/s) |
