# Supplementary material for: Defining frailty using a modified Fried’s Frailty Phenotype in a Southern African context
Source: PLoS One. 2026 Feb 4;21(2):e0340723. doi: 10.1371/journal.pone.0340723 (PMC12872031; doi:10.1371/journal.pone.0340723)
Supplement: S5 Table — (DOCX) [file pone.0340723.s007.docx]

| **Anthropometry** | | | | |
| --- | --- | --- | --- | --- |
| **001** | *DATE1* | Date of assessment (dd/mm/yyyy) | □□/□□/□□□□ | |
| **002** | *INTID* | Interviewer ID | □□□ | |
| **003**  **004** | *HTSIT* | Sitting Height | **1^st^**□□□.□cm | **2^nd^**□□□.□cm |
| **005**  **006** | *CHHT* | Chair Height | **1^st^**□□□.□cm | **2^nd^**□□□.□cm |
| **007**  **008** | *HT* | Standing Height | 1^st^ □□□.□cm | 2^nd^ □□□.□cm |
| **009** | *AVH* | Average (mean) standing height | □□□.□cm | |
| **010**  **011** | *WGT* | Weight | 1^st^ □□.□/kg | 2^nd^ □□.□kg |
| **012** | *AVW* | Average (mean) weight | □□.□kg | |
| **013**  **014** | *HT* | Knee height | **1^st^**□□□.□cm | **2^nd^**□□□.□cm |
| **015**  **016** | *WTR* | Wall to tragus | **1^st^**□□□.□cm | **2^nd^**□□□.□cm |
| **017**  **018** | *CIRC* | Mid-upper arm circumference  (non-dominant arm) | **1^st^**□□□.□cm | **2^nd^**□□□.□cm |
| **019**  **020** | *CIRC* | Waist circumference | **1^st^**□□□.□cm | **2^nd^**□□□.□cm |
| **021**  **022** | *CIRC* | Hip circumference | **1^st^**□□□.□cm | **2^nd^**□□□.□cm |
| **023** | *BVI* | BVI % **visceral fat** | | □□.□% |

| **SPPB: Chair Stands** | | | | | | | | |
| --- | --- | --- | --- | --- | --- | --- | --- | --- |
| **024** | *STS* | | A. Safe to stand x 5 | | | Yes No | | |
| **025** | *TTS* | | B. Time to stand | | | 1^st^ □□□ seconds | | 2^nd^ □□□ seconds |
| **026** | *FAI* | | C. If did not attempt or failed, provide reason: | | | Tried but unable  Unable to stand unassisted  Not attempted, you felt unsafe    Not attempted, participant felt unsafe  Unable to understand instructions  Refused  Other (Specify): | | |
| **SPPB: Balancing testing** | | | | | | | | |
| **027** | | *FTG* | | Feet together | Able to complete 10 seconds  Not able to complete 10 seconds | | | |
| **028** | | *SET* | | Semi-tandem | Able to complete 10 seconds  Not able to complete 10 seconds | | | |
| **029** | | *TAM* | | Full tandems | Able to complete 10 seconds  Not able to complete 10 seconds | | | |
| **SPPB: Walk speed (over 4 metres)** | | | | | | | | |
| **030** | | *NWK* | | Normal walk [see SOP] Did respondent complete the walk at usual pace? | | | Yes No, refused  No, cannot walk even with support | |
| **031** | | *NW1* | | Normal walk. Time at 4 metres, First attempt: | | | □□□ seconds | |
| **032** | | *WA1* | | Walking aid used for first attempt | | | Yes No | |
| **033** | | *NW2* | | Normal walk: Time at 4 metres, Second attempt | | | □□□ seconds | |
| **034** | | *WA2* | | Walking aid used for second attempt | | | Yes No | |

| **Grip Strength** | | | | |
| --- | --- | --- | --- | --- |
| **035** | *OID* | Operator | | □□□ |
| **036** | *GPDT* | Date | | □□/□□/□□□□ |
| **037** | *CON1* | Contraindication to measurement on **right** hand *(Have you had any surgery on your right arm, hand or wrist in the last 3 months OR arthritis or pain in your right hand or wrist)?* | | Yes□ No□ |
| **038** | *CON2* | Contraindication to measurement on **left** hand *(Have you had any surgery on your left arm, hand or wrist in the last 3 months OR arthritis or pain in your left hand or wrist)?* | | Yes□ No□ |
| **039** | *DOM* | Hand Dominance | Right-handed □  Left-handed □  Ambidextrous□ | |
| **040** | *COM* | Did respondent complete the grip strength test? | Yes □  No, refused □  No, unable to understand instructions □  No, too weak to perform test □ | |
|  | *RES* | **Right** | **Left** | |
| **041**  **042** | **1^st^ Measurement** | □□.□kg | □□.□kg | |
| **043**  **044** | **2^nd^ Measurement** | □□.□kg | □□.□kg | |
| **045**  **046** | **3^rd^ Measurement** | □□.□kg | □□.□kg | |
| **047** | *FINF* | Greatest of 6 measurements | □□.□kg | |
| **048** | *COM1* | Comments - please provide detail of contraindications or reasons grip strength not performed: | | |

| **GALS Examination Questions** | | | |
| --- | --- | --- | --- |
| **049** | *SYMS* | Any MSK symptoms? | joint pain - Yes No  muscle pain - Yes No  problem dressing - Yes No |
| **050** | *GAI* | Gait | Normal appearance - Yes No  Normal movement - Yes No |
| **051** | *ARM* | Arms | Normal appearance - Yes No  Normal movement - Yes No |
| **052** | *LEG* | Legs | Normal appearance - Yes No  Normal movement - Yes No |
| **053** | *SPIN* | Spine | Normal appearance - Yes No  Normal movement - Yes No |
| **054** | PDET | Please provide details of any abnormality identified. Please include the side for any limb affected: | |

| **Blood Pressure (BP)** | | | |
| --- | --- | --- | --- |
| **055** | *FATE* | BP cuff size | **SMALL MEDIUM**  **LARGE** |
| **056** | *BP1* | BP reading 1 | /mmHg |
| **057** | *BP2* | BP reading 2 | /mmHg |
| **058** | *BP3* | BP reading 3 | /mmHg |
| **059** | *LBP* | lowest BP reading | /mmHg |
| **060** | *BMED* | Already taking BP medication | yes no don’t know |
| **061** | *BACT* | Action taken | 0=none 1=physician referral 2=PI informed |
| **062** | *BPCO* | Comments |  |

| **Blood Glucose** | | | |
| --- | --- | --- | --- |
| **063** | *GSDT* | Date blood sample collected  (dd/mm/yyyy) | // |
| **064** | *GTME* | Time specimen collected (24h clock) | : |
| **065** | *FASD* | Date participant last ate  (dd/mm/yyyy) | // |
| **066** | *FATE* | Time participant last ate  (use 24-hour clock) | : |
| **067** | *GLUC* | Blood glucose reading | .mmol/L |
| If any abnormality is identified please refer to study doctor who will complete regional examination and refer participant for further evaluation and management as appropriate. | | | |

| **Vision Assessment:** | | | | |
| --- | --- | --- | --- | --- |
| **068** |  | Date of assessment | // | |
| **069** |  | ID of vision tester | Distance  Near | |
| **1. Distance Vision using Peek vision test at 2m (logMar)** | | | | |
| **070 -**  **079** | *If refused, fill in 999* | Uncorrected Distance Vision (logMar) for each eye separately | Right eye *(select + or -)* | Left eye *(select + or -)* |
|  |  |  | +/- . | +/-. |
|  |  | Corrected Distance Vision (logMar) for each eye separately (SKIP or serial 8s if no distance correction available) | +/- . | +/-. |
|  |  | Corrected with *(several may apply)*  Spectacles  Contact lenses  Other *(specify)*  Not applicable | 1  2  3  8 | 1  2  3  8 |
| **2. Near Vision using tumbling E near vision test at 40cm (Near [Times Roman printer’s points])** | | | | |
| **080** | *If refused, fill in 999* | Uncorrected Near Vision (Times Roman Printer’s Points) both eyes open at 40cm | N. | |
| **081** |  | Corrected Near Vision (Times Roman Printer’s Points) both eyes open at 40cm | N. | |
| **082**  **083** |  | Corrected with *(several may apply)*  Spectacles  Contact lenses  Other *(specify)*  Not applicable | 1  2  3  8 | 1  2  3  8 |
